# A Portable Spectrometric System for Quantitative Prediction of the Soluble Solids Content of Apples with a Pre-calibrated Multispectral Sensor Chipset

**DOI:** 10.3390/s20205883

**Published:** 2020-10-17

**Authors:** Nhut-Thanh Tran, Masayuki Fukuzawa

**Affiliations:** 1 Graduate School of Science and Technology, Kyoto Institute of Technology, Matsugasaki, Sakyo-ku, Kyoto 606-8585, Japan; 2 Department of Automation Technology, College of Engineering Technology, Can Tho University, Can Tho 900100, Vietnam; nhutthanh@ctu.edu.vn

**Keywords:** internal fruit quality, multispectral sensor, quantitative prediction, reproductive alignment, system manufacturability, soluble solids content

## Abstract

A portable spectrometric system for nondestructive assessment of the soluble solids content (SSC) of fruits for practical applications has been proposed and its performance has been examined by an experiment on quantitative prediction of the SSC of apples. Although the spectroscopic technique is a powerful tool for predicting the internal qualities of fruits, its practical applications are limited due to its high cost and complexity. In the proposed system, the spectra of apples were collected by a simple optical setup with a cheap pre-calibrated multispectral chipset. An optimal multiple linear regression model with five wavebands at 900, 760, 730, 680, and 535 nm revealed the best performance with the coefficient of determination of prediction and the root mean square error of prediction of 0.861 and 0.403 °Brix, respectively, which was comparable to that of the previous studies using dispersive spectrometers. Compared with previously reported systems using discrete filters or light emitting diodes, the proposed system was superior in terms of manufacturability and reproducibility. The experimental results confirmed that the proposed system had a considerable potential for practical, cost-effective applications of the SSC prediction, not only for apples but also for other fruits.

## 1. Introduction

Although internal fruit qualities such as sugar content, firmness, and ripeness strongly affect consumer satisfaction, they are sometimes difficult to predict only by visual observation. Internal qualities do not always correspond to external ones such as color, size, shape, and so on. Therefore, it is important to evaluate the internal quality of fruits before selling them to customers. Many techniques have been successfully applied to evaluate nondestructively fruit quality such as ultrasound, visible and near-infrared (VIS–NIR) spectroscopy, hyperspectral imaging, machine vision, impact analysis, and so on [1]. In these techniques, VIS–NIR spectroscopy combined with chemometrics is the most popular one and it has been successfully implemented to measure the internal quality of fruits [2].

There are several VIS–NIR spectroscopy techniques such as dispersive, Fourier transform, filter-based, and light emitting diode (LED)-based ones [3,4]. Dispersive and Fourier transform techniques are based on a special optical system such as grading and interferometry. Their complexity is disadvantageous in terms of fabrication cost, but they have a certain advantage of high spectral resolution over a wide range. Therefore, they are favorable at the investigation stage in the laboratory where critical wavebands are uncertain. On the other hand, simple optical systems such as filter- or LED-based devices are preferred for practical applications in the field where only a few wavebands are relevant but portability and low system cost are strongly demanded.

In the case of filter- or LED-based devices, the component of each waveband is separated and consequently has a different optical path and spectral sensitivity. However, they should be kept equivalent over all wavebands to apply spectroscopy for chemometrics. Therefore, alignment of optical path and sensitivity calibration are very important for these devices. If the device consists of discrete elements of LED, filter, and photodiode, the alignment and calibration become more essential for system performance [5] and, in some cases, they are against system manufacturability and reproducibility.

Several previous studies tried to fabricate the portable spectrometric system to evaluate the quality of fruits in the field by adopting filter- or LED-based devices. Saeed et al. [6] developed a multiband sensor system to detect oil palm fresh fruit bunch maturity. They used four discrete illuminator-detector sets aligned in parallel to obtain reflectance spectra with 60 cm of working distance. Giovenzana et al. [7] tested the ability of an LED-based device for rapid estimation of white grape ripeness. They used an eight-arm optical fiber to couple four LEDs and four photodiodes. A five-band portable sensor was investigated by Jam and Chia [8] to classify the internal quality of pineapples. They used one photodiode and an integrated light source with five near-infrared LEDs mounted on a special pentagonal fixture. These previous studies have successfully demonstrated the potential of filter- or LED-based devices for checking the quality of fruits. However, these studies mainly focused on qualitative assessment such as classification. Furthermore, these systems required special treatments such as a customized fixture or optical parts and individual alignment for all discrete components to keep an equivalent optical path, which may limit system manufacturability and reproducibility. As for commercially available systems, there are various products based on filter- or LED-based techniques such as a DA-meter (T.R. Turoni Srl, Forli, Italy) [9], but the dispersive spectrometer is still adopted into conventional apparatus such as the F-750 Produce Quality Meter (Felix Instruments, Washington, USA) [10], which may imply that there is still plenty of room for improvement in filter- or LED-based devices.

In this study, we proposed a simple and portable spectrometric system for quantitative prediction of the soluble solids content (SSC) of fruits with a pre-calibrated multispectral sensor chipset (AS7265x). The chipset has 18 channels of wavebands covering a visible to shortwave near-infrared (VIS–SWNIR) range from 410 to 940 nm, which is suitable for evaluating the internal qualities of fruits because of the higher penetration ability [11,12]. The equivalent optical path was obtained with a simple optical setup utilizing chipset characteristics. The performance of the proposed system was evaluated by quantitative prediction of the SSC for apples with some standard regression models and compared with that of a commercial dispersive spectrometer. There are some discussions on the manufacturability and reproducibility of the system, which are important for practical applications.

## 2. Materials and Methods

### 2.1. Sample Preparation

A total of 80 *Kiou* apples was purchased from a supermarket in Kyoto, Japan. The *Kiou* apples have yellow peel, white flesh, and a round to round-heart shape. They were mainly grown in Aomori Prefecture, Japan. All selected apples for this study had no external damage and were of regular size and shape. The average weight of each apple was around 300 g. These apples were then kept at room temperature (25 °C) for 24 hours before conducting the experiment. All spectral measurements and destructive tests were performed at room temperature.

### 2.2. Experimental Setup

**Figure 1:**
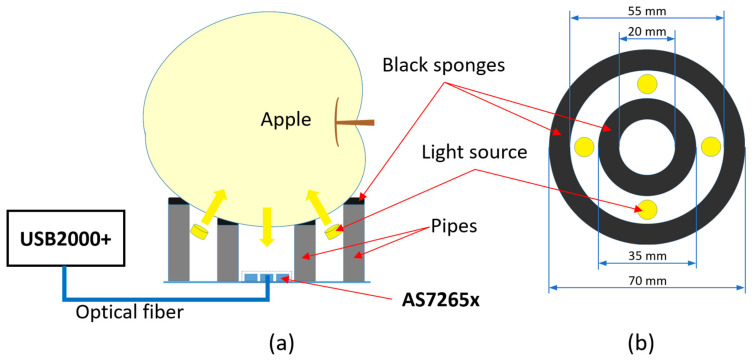
Schematic diagram of the experimental setup in the interactance measurement (**a**) and the dimensions of sponges (**b**).

The experimental setup in this study was designed as a prototype of a portable system with high manufacturability and reproducibility for rapid prediction of internal fruit qualities in the field. Three different measurement modes (transmittance, reflectance, and interactance) are usually used to evaluate fruit quality. Among these three modes, the interactance mode was selected in this study. In the interactance mode, the light source and detector are positioned on the same side of a sample, and they are isolated by a light shield to prevent specular reflection [3]. It is advantageous to the portable system because the measurement can be performed by placing the sample from above. The interactance mode is considered the best for predicting the internal quality of fruits and vegetables [13]. The schematic diagram of the experimental setup is depicted in Figure 1.

The light source consisted of four 3.6 W halogen bulbs from the Ohm Electric company and was driven by a regulated power supply. They were isolated from the detector by two cylindrical light shields with black paper (20 and 27 mm in height), shown in Figure 1a. Two rings of black foam sponge were attached on the top of the cylindrical pipes to hold apples, which also acted as part of the light shields. The dimensions of sponges are shown in Figure 1b.

The detector adopted in this study was the AS7265x sensor chipset. It consists of three compact sensor chips and each chip integrates a built-in aperture, on-device optical filters, and a photodiode array with a precise alignment. The chipset covers 18 wavebands centered at 410, 435, 460, 485, 510, 535, 560, 585, 610, 645, 680, 705, 730, 760, 810, 860, 900, and 940 nm with the 20 nm of unified full wave at half maximum (FWHM). The optical characteristics are optimized for diffused light with a wide aperture and the spectral sensitivity was pre-calibrated [14]. In order to ensure the equivalent optical path by utilizing the wide aperture of the chipset, the working distance (20 mm) was designed as long as the chipset size and no external lenses or mirrors were used.

Additionally, a small light-incident part of the optical fiber with a wide aperture was arranged at the center of the chipset so that it would not conflict to any other parts. It is not a part of the portable system prototype, but it enables us to in situ monitor the interference spectra to be measured by the prototype. A commercial dispersive spectrometer (USB2000+) was used for spectra monitoring, which equips a 2048-pixel linear image sensor (SONY ILX511B), and it covers 200–1100 nm with a spectral resolution of 0.3 nm.

### 2.3. Spectra Acquisition

The spectral data of the AS7265x chipset were collected by ams_Spectral_Sensor_Dashboard software provided by the company ams AG (Premstaetten, Austria). The OceanView software was used to get spectral data of the USB2000+ spectrometer. The integration time of each scan was 400 ms for the AS7265x and 200 ms for the USB2000+. All spectral measurements were conducted in a dark-room condition to prevent any interactance to the apples except for the built-in light source.

Two samples on the opposite sides of each apple were collected. The apples were positioned on the sponges with horizontal stem–calyx orientation and a portion of the apples was illuminated by the four bulbs. After interacting with the apple tissue, the diffused light was introduced to the chipset. The intensities of the diffused light from the apples detected by the chipset were considered as raw spectra. The spectra of each sample were calculated by averaging 20 scans, noted as ${\hat{I}}_{\mathrm{raw}}\left(\lambda_{i}\right)$. A standard 18% gray reflector plate was placed 20 mm from the top of the sponges to obtain averaged reference spectra as ${\hat{I}}_{\mathrm{reference}}\left(\lambda_{i}\right)$. The reference spectra were collected for every four samples to confirm its stability. The relative reflectance spectra, $R\left(\lambda_{i}\right),$ and absorbance spectra, $A\left(\lambda_{i}\right),$ were then calculated by Equations (1) and (2).

(1) $$R\left(\lambda_{i}\right)=\frac{{\hat{I}}_{raw}\left(\lambda_{i}\right)}{{\hat{I}}_{reference}\left(\lambda_{i}\right)}$$

(2) $$A\left(\lambda_{i}\right)=\;log_{10}\left(\frac{1}{R\left(\lambda_{i}\right)}\right)$$

where $\lambda_{i}$ are the *i*th wavelengths of the USB2000+ spectrometer or the AS7265x sensor. The absorbance spectra were then used to build calibration and prediction models.

### 2.4. Reference Analysis

After spectral measurements, to extract apple juice for each sample, in the same area of each spectral measurement, the apple was cut about 5 mm in depth from the peel due to the light penetration of apples [12]. The apple juice was then measured three times by a portable Brix-acidity meter (model PAL-BX|ACID F5 Master Kit, Atago Co., Ltd., Tokyo, Japan). This meter has a Brix measurement range from 0 to 60 °Brix with automatic temperature compensation; the resolution and accuracy of this meter are 0.1 and ± 0.2 °Brix, respectively. The average value of three measurements was considered as a final value for each sample.

### 2.5. Statistical Analysis

In this study, multiple linear regression (MLR) and partial least squares regression (PLSR) were selected to build regression models. PLSR is well known as a standard analysis technique in spectrometry to obtain a calibration curve from the spectral data where the number of wavebands sometimes exceeds the number of samples. PLSR can deal with data including multicollinearity effectively by projecting the original data into a new latent structure with less correlation [15]. It was applied to compare the performance between AS7265x and USB2000+. Although MLR is the essential linear regression model without any projection, it is applicable and generally applied in analyzing spectral data from filter- or LED-based devices where the number of wavebands are limited and less than the number of samples. It was adopted only to obtain an optimal regression model for AS7265x with an optimal combination of a few wavebands. These models were implemented by the Statistics and Machine Learning Toolbox of MATLAB version 9.7 software (The MathWorks, Inc., USA).

The optimal waveband combination was selected mainly based on the sequential forward selection (SFS) algorithm with some simplification. It begun with finding the best one-waveband by calculating the correlation coefficients between spectral data and Brix values of the calibration data with MLR for each waveband. Consequently, the best two-waveband combination was determined by examining the correlation for all possible (17) two-waveband combinations of the best one-waveband and another one. The best *k*-waveband combination was repeatedly determined while increasing *k* until its maximum (18), rather than stopping when the performance increase was saturated, which is found in conventional SFS. The optimal waveband combination was selected retrospectively by comparing the prediction performance of the MLR model among all 18 waveband combinations. Some simplified SFS algorithms were adopted even in previous studies to predict the internal quality of fruits [16,17] because this is much faster than an exhaustive search algorithm and is known to balance between model complexity and performance [18].

Performances of the models were compared in terms of coefficient of determination ($R^{2}$) and root mean square error (RMSE) for both calibration model ($R_{c}^{2}$, RMSEC) and prediction model ($R_{p}^{2}$, RMSEP) using Equations (3)–(6).

(3) $$R_{c}^{2}=1-\frac{\sum_{i=1}^{n_{c}}{\left(y_{i}-{\hat{y}}_{i}\right)}^{2}}{\sum_{i=1}^{n_{c}}{\left(y_{i}-{\bar{y}}_{i}\right)}^{2}}$$

(4) $$RMSEC=\sqrt{\frac{1}{n_{c}}\overset{n_{c}}{\underset{i=1}{\sum }}{\left({\hat{y}}_{i}-y_{i}\right)}^{2}}$$

(5) $$R_{p}^{2}=1-\frac{\sum_{k=1}^{n_{p}}{\left(y_{k}-{\hat{y}}_{k}\right)}^{2}}{\sum_{k=1}^{n_{p}}{\left(y_{k}-{\bar{y}}_{k}\right)}^{2}}$$

(6) $$RMSEP=\sqrt{\frac{1}{n_{p}}\overset{n_{p}}{\underset{k=1}{\sum }}{\left({\hat{y}}_{k}-\;y_{k}\right)}^{2}}$$

where ${\hat{y}}_{i},{\hat{y}}_{k}$ denote the predicted Brix value of the *i*th sample in the calibration set and the *k*th sample in the prediction set; $y_{i},\;y_{k}$ are the measured (reference) Brix value of the *i*th sample in the calibration set and the *k*th sample in the prediction set; $n_{c},{\;n}_{p}$ are the number of samples in the calibration set and prediction set. Generally, a better model should have higher values of $R_{c}^{2}$ and $R_{p}^{2}$, and lower values of RMSEC and RMSEP.

## 3. Results

### 3.1. Spectral Responses

**Figure 2:**
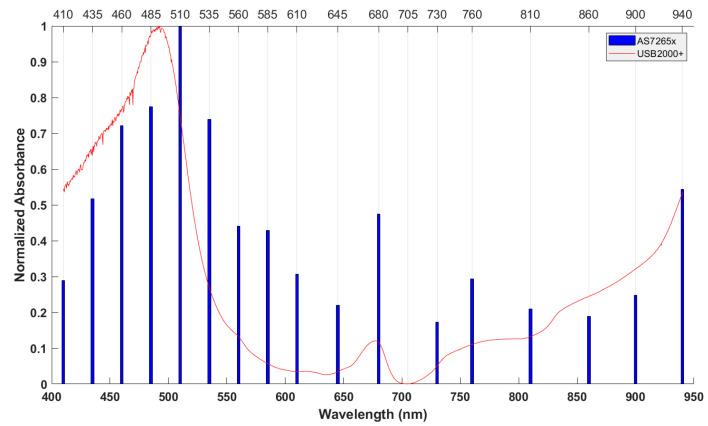
Typical absorbance spectra obtained by AS7265x and USB2000+.

Figure 2 shows typical absorbance spectra $A\left(\lambda_{i}\right)$ of apples obtained by an AS7265x chipset and USB2000+ spectrometer. There were two absorbance peaks in the visible region, one peak around 500 nm due to anthocyanin content, the other around 680 nm related to chlorophyll content. The spectra had a trough at 705 nm and increased gradually in the near-infrared region, which is related to the 4th and 3rd overtone C-H bonds and the 3rd overtone O-H bonds. The absorbance trend of the AS7265x chipset was similar to that of the USB2000+ spectrometer.

### 3.2. PLSR Models for USB2000+ and AS7265x

After removing a few outliers, 148 samples were divided into two different sets by sample set portioning based on the joint X–Y distance (SPXY) method: 100 samples for the calibration set and 48 samples for the prediction set. Statistical information of calibration and prediction sets are shown in Table 1.

PLSR models were developed to predict Brix values of the samples using all 18 wavebands of the AS7265x sensor. In order to compare fairly the performance of the AS7265x, 1567 wavelengths from 410 to 940 nm of USB2000+ were used. The optimal number of latent variables in these models was chosen based on mean squared prediction error by cross-validation using 10-fold cross-validation. Absorbance data of USB2000+ were pre-processed by the Savitzky–Golay smoothing method. Calibration and prediction results of PLSR models are shown in Table 2.

The PLSR models gave good results for both calibration and prediction sets, the *R^2^* values were greater than 0.803, and the RMSE values were less than 0.599 °Brix. In comparison of the prediction results, the coefficient of determination and the root mean square error of the USB2000+ data were slightly higher than those of the AS7265x data.

**Figure 3:**
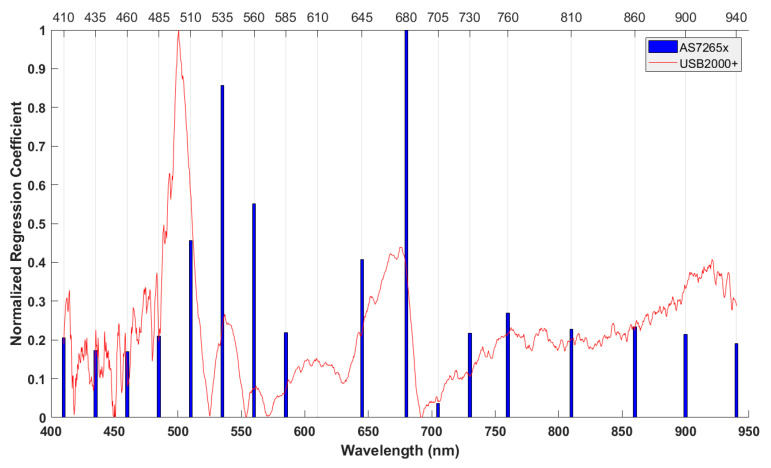
Regression coefficients of calibration data for USB2000+ and AS7265x.

Figure 3 shows absolute values of the regression coefficients of PLSR models using four latent variables for USB2000+ data and two latent variables for AS7265x data. The wavelengths with high regression coefficient occurred at around 500, 680, and 900 nm for USB2000+ data, whereas they were at 535 and 680 nm for AS7265x data. These wavelengths had greatly contributed to PLSR models.

### 3.3. Optimal MLR Model for AS7265x

**Figure 4:**
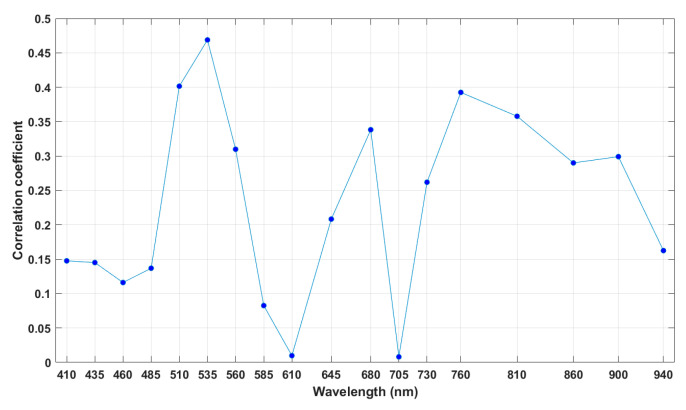
Correlation coefficients of calibration data for all 18 wavebands.

In the first step of the waveband selection process using the SFS algorithm, correlation coefficients between spectral data and Brix values of calibration data for each waveband of AS7265x chipset were calculated. These coefficients are shown in Figure 4.

In Figure 4, the highest correlation coefficient occurred at 535 nm. Therefore, the waveband at 535 nm was considered as the best one-waveband. The correlation coefficients of the combinations were calculated as the above descriptions. Finally, the combination of 18-waveband was arranged in order of 535, 680, 900, 760, 730, 460, 610, 510, 435, 705, 485, 410, 940, 860, 645, 585, 560, and 810 nm.

The performance of MLR models using different waveband combinations is shown in Table 3. From this table, calibration performance of the models was gradually improved (higher $R_{c}^{2}$ value and lower RMSEC value) when the number of wavebands in the combination was increased, but the prediction performance of the models did not follow that trend.

**Figure 5:**
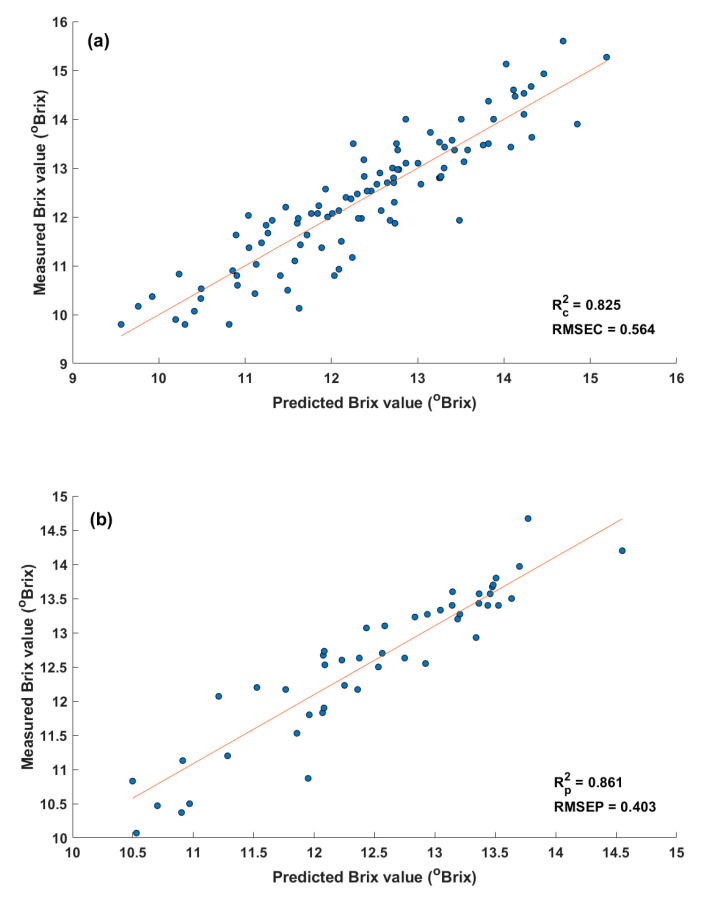
Relationships between measured and predicted Brix values using five selected wavebands for (**a**) calibration data and (**b**) prediction data.

According to Table 3, MLR models using the five-waveband combination (No. 5) and seven-waveband combination (No. 7) gave similarly good prediction performances. This fact represents that the contribution of two wavebands at 460 and 610 nm is not significant for improving performance. Therefore, model No. 5 was better than model No. 7 in terms of model complexity. Compared with the model using the four-waveband combination (No. 4), model No. 5 became more complex (more than one waveband at 730 nm), but the prediction performance of model No. 5 was superior to that of model No. 4. Moreover, the waveband at 730 nm is related to the absorption band of carbohydrate, which is a main component of the SSC of apples. Thus, there is no advantage to reduce model complexity by removing the 730 nm wavelength from the model. According to the reasons above, the waveband combination of the model No. 5 was selected as an optimal waveband combination. These five wavebands also corresponded to wavelengths with a high regression coefficient from the PLSR models (Figure 3). Two scatter plots of the measured (reference) and predicted Brix values of the MLR model using the five-waveband combination are depicted in Figure 5. 

In comparison with results of the PLSR model using all 18 wavebands in Table 2, the performance of the MLR model using optimal wavebands from the simplified SFS algorithm was slightly improved. The $R_{p}^{2}$ value was increased from 0.852 to 0.861, and the RMSEP value was decreased from 0.416 to 0.403 °Brix. Since the MLR model gave an equivalent and partly better performance to that of the PLSR model, it could be confirmed that multicollinearity was not harmful to prediction performance in the optimized MLR model. Moreover, calibration and prediction results of the MLR model using five optimal wavebands were roughly equal to those of the PLSR model for USB2000+ data for predicting the SSC of apples.

## 4. Discussion

According to the resulting performance of PLSR models, the proposed system revealed comparable spectrometric performance to that of the dispersive spectrometer USB2000+. Since the price of the AS7265x chipset is about one tenth as much as that of USB2000+, the proposed system can be manufactured in a very cost-effective way. Additionally, it is noted here that the AS7265x chipset equips controllable current drivers to drive the light source and built-in temperature sensors to compensate the waveband drift. Although they were not necessary in this study, they are very valuable for the portable spectrometric system used in the field.

The SSC prediction performance of the system was $R_{p}^{2}\;$= 0.861, RMSEP = 0.403 °Brix, by using the optimal MLR model with the five effective wavebands at 900, 760, 730, 680, and 535 nm. The SSC prediction performance was comparable to that of previous studies on apples with dispersive spectrometers when considering both $R_{p}^{2}$ and RMSEP [19,20,21,22,23]. The appropriateness of selected wavebands was confirmed not only by corresponding absorbance origins but also because they have been utilized in many previous studies for evaluating the SSC or sugar content of fruits. The two wavebands at 535 and 680 nm are related to major pigments (anthocyanin and chlorophyll) of apples, and these pigments of apples will change with change of its ripeness. The region around 535 and 680 nm was successfully used to predict the SSC of apples [24,25,26,27] and other fruits [28,29,30]. The waveband at 760 nm (mainly due to water absorption) was useful for predicting the SSC of apples because water content is inversely proportional to SSC in fruits. A strong correlation between absorbance and Brix value around 760 nm was also found by Guthrie et al. [31] and Rajkumar et al. [28]. The absorption at 730 and 900 nm are related to carbohydrates and these wavebands were applied to determine the concentration of sugar solutions [32]. The waveband around 900 nm was successfully implemented to build a universal model for predicting the SSC of three fruit species (apple, pear, and peach) [33]. Several previous reports also used around 900 nm to predict the SSC of apples [25,26] and other fruits [17,29,34,35]. This many reports with similar wavebands not only ensure the appropriateness of the selected wavebands but may also imply the system potential for application to other fruits.

The main difference between the proposed and previous systems is the conciseness of the optical system. By adopting the compact multispectral sensor chipset, the number of system components was drastically reduced and individual sensitivity calibration was not required. No customized fixtures or optical parts were required by the design of the simple optical setup utilizing the chipset characteristics. It is very favorable to system manufacturability because it requires only a few generic and cheap components such as a halogen lamp and paper light shield. Furthermore, such a simple setup consequently eliminates the need for individual alignment, which strongly depends on system reproducibility. Therefore, manufacturability and reproducibility of the proposed system were obviously superior to those of previous systems based on discrete components [5,6,7], which also implies the potential of the proposed system for a wide variety of cost-effective applications in the field.

## 5. Conclusions

In this study, we have successfully developed a portable spectrometric system that could apply for practical application in the field to quantitatively predict the SSC of fruits. By combining a simple optical setup based on a pre-calibrated multispectral sensor chipset (AS7265x) and its optimal MLR model, good spectrometric performance was achieved at only one tenth the cost of a commercial dispersive spectrometer. The system revealed good performance on quantitative SSC prediction of apples, which is comparable to that in previous studies with dispersive spectrometers. System manufacturability and reproducibility were superior to those of previously reported systems with a simplified optical system based on discrete devices because no special treatments, such as customized fixtures or optical parts, and individual alignments, were required in the system. Since the wavebands in which the system achieved enough good performances are also relevant to many kinds of fruits other than apples, the system could have a significant potential for a wide variety of practical cost-effective applications of internal quality assessment, not only for apples but also for other fruits.

## Figures and Tables

**Table 1:** Statistics of calibration and prediction sets.

Items	Calibration	Prediction
Number of samples	100	48
Range (°Brix)	9.8–15.6	10.0–14.7
Mean value (°Brix)	12.55	12.38
Standard deviation (°Brix)	1.33	1.14

**Table 2:** Performance of partial least squares regression (PLSR) models for USB2000+ and AS7265x.

Data	Latent Variable	Calibration		Prediction	
		R_c_^2^	RMSEC	R_p_^2^	RMSEP
USB2000+	4	0.816	0.568	0.876	0.398
AS7265x	2	0.803	0.599	0.852	0.416

**Table 3:** Performance of multiple linear regression (MLR) models with different waveband combinations.

No.	Selected Waveband (nm)	Calibration		Prediction	
		R_c_^2^	RMSEC	R_p_^2^	RMSEP
1	535	0.469	0.984	0.406	0.833
2	535, 680	0.775	0.641	0.769	0.519
3	535, 680, 900	0.808	0.591	0.846	0.424
4	535, 680, 900, 760	0.813	0.583	0.856	0.409
**5**	**535, 680, 900, 760, 730**	**0.825**	**0.564**	**0.861**	**0.403**
6	535, 680, 900, 760, 730, 460	0.827	0.562	0.849	0.420
7	535, 680, 900, 760, 730, 460, 610	0.829	0.558	0.864	0.398
8	535, 680, 900, 760, 730, 460, 610, 510	0.837	0.545	0.834	0.441
9	535, 680, 900, 760, 730, 460, 610, 510, 435	0.843	0.536	0.808	0.473
10	535, 680, 900, 760, 730, 460, 610, 510, 435, 705	0.844	0.534	0.808	0.474
…	…	…	…	…	…
18	All 18 wavebands	0.862	0.502	0.781	0.505
